# *Posidonia oceanica* (L.) Delile in Focus: In Vitro and In Vivo Evidence for Biomedical Potential

**DOI:** 10.3390/ijms27041727

**Published:** 2026-02-11

**Authors:** Marzia Vasarri, Lucia De Marchi, Carlo Pretti, Donatella Degl’Innocenti

**Affiliations:** 1 Department of Experimental and Clinical Biomedical Sciences, University of Florence, 50134 Florence, Italy; donatella.deglinnocenti@unifi.it; 2 Department of Veterinary Sciences, University of Pisa, 56124 Pisa, Italy; lucia.demarchi@unipi.it (L.D.M.); carlo.pretti@unipi.it (C.P.); 3 Interuniversity Center of Marine Biology and Applied Ecology “G. Bacci” (CIBM), Viale N. Sauro 4, 57128 Leghorn, Italy

**Keywords:** *Posidonia oceanica*, marine phytotherapy, bioactive compounds, biomedical uses

## Abstract

*Posidonia oceanica* (L.) Delile, an endemic seagrass of the Mediterranean Sea, has been increasingly recognized not only for its ecological significance but also for its potential as a source of bioactive compounds in human health. Over the past decade, scientific studies have identified diverse constituents of *P. oceanica*, including polyphenols, peptides, and polysaccharides, which exhibit antioxidant, anti-inflammatory, cytotoxic, and metabolic regulatory activities. Evidence from in vitro and in vivo models demonstrates its ability to influence key cellular processes such as apoptosis, autophagy, and enzyme inhibition, suggesting therapeutic promise in cancer, skin aging, inflammatory conditions, and metabolic disorders like type 2 diabetes. Recent advances in delivery technologies, such as nanoparticles, micelles, and liposomes, have improved the stability and bioavailability of *P. oceanica* extracts, enhancing their potential application in pharmaceuticals and cosmeceuticals. Additionally, its antimicrobial and antibiofilm properties suggest applications in food preservation and infection control. By bridging traditional uses with modern scientific validation, *P. oceanica* exemplifies the emerging potential of marine phytotherapy. As interest grows in nature-derived therapeutics, further research is essential to translate these preclinical findings into clinical applications while ensuring sustainable management and the conservation of this valuable marine resource.

## 1. Introduction

*Posidonia oceanica* (L.) Delile, commonly known as “Neptune grass”, is a seagrass species endemic to the Mediterranean Sea that forms vast underwater meadows covering roughly 1.5% of the Mediterranean seabed. These meadows represent one of the most important and productive coastal ecosystems in the region, characterized by a remarkable capacity to support marine biodiversity and maintain environmental balance [1].

As a marine angiosperm uniquely adapted to saline and submerged conditions, *P. oceanica* has evolved a suite of morphological and physiological traits that enable it to thrive in dynamic coastal environments, playing a critical role in stabilizing sediments and maintaining water quality. Its dense underwater meadows act as nursery grounds and habitats for a wide variety of marine species, ranging from invertebrates and fish to endangered sea turtles and marine mammals, thereby contributing to the complex food webs and ecological networks of the Mediterranean Sea [2].

Beyond its biological importance, *P. oceanica* performs essential environmental functions, including oxygen production, sediment stabilization, and carbon sequestration. These ecological services are particularly relevant in the context of climate change and increasing human pressure, highlighting the need for conservation practices and sustainable management [3]. Recognizing its ecological value, *P. oceanica* is protected under various environmental regulations, including the European Union’s Habitats Directive, which mandates the preservation and sustainable management of these critical habitats [4].

**Figure 1:**
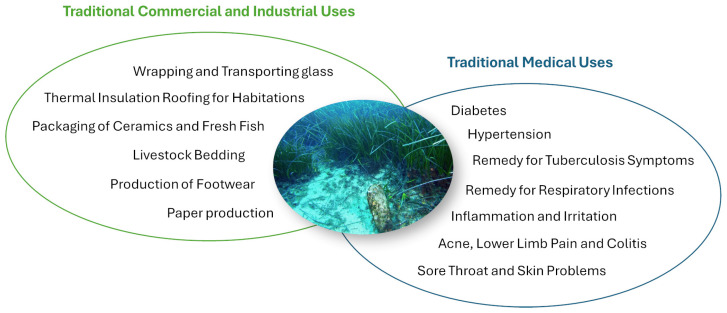
Schematic representation of the traditional uses of the marine plant *P. oceanica* in commercial, industrial and healthcare sectors.

Historically, *P. oceanica* has also maintained a significant, albeit less explored, role in human culture and industry. Its fibrous leaves have been used for construction and insulation materials, while its residues have been utilized in agriculture as fertilizers. Traditional medicinal practices have also incorporated *P. oceanica* for its purported health benefits, illustrating the multifaceted relationship between humans and this remarkable marine angiosperm (Figure 1) [5].

**Figure 2:**
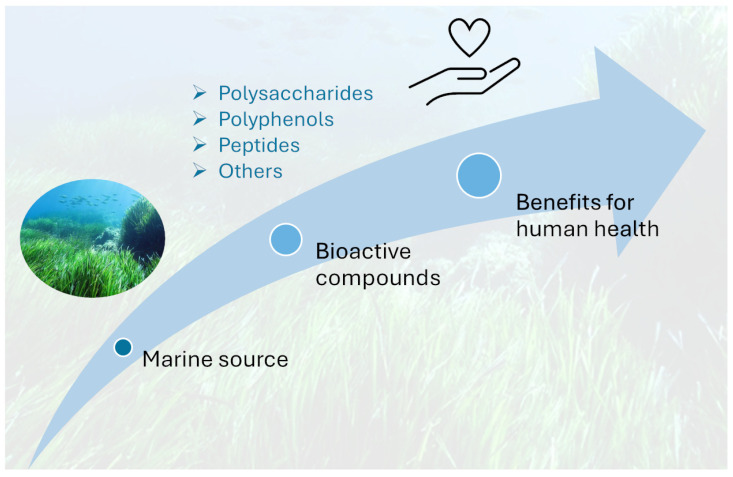
The seagrass *P. oceanica* as a marine source of bioactive compounds with potential applications for human health.

In recent decades, scientific research has increasingly focused on the phytochemical composition of *P. oceanica*, revealing a complex matrix of bioactive molecules such as polyphenols, peptides, polysaccharides, and other secondary metabolites. These compounds have demonstrated a broad spectrum of biological activities, including potent antioxidant and anti-inflammatory effects, as well as cytotoxic and anti-migratory properties relevant to cancer cell biology. The interplay of these activities suggests that *P. oceanica* extracts may modulate critical cellular processes such as apoptosis, autophagy, and enzyme regulation, which are central to the pathogenesis of various diseases. Moreover, emerging evidence highlights the potential role of *P. oceanica* in metabolic health, showing promising glucose-lowering, anti-glycation, and lipid-regulating effects that could contribute to the management of diabetes and related metabolic disorders. The combination of ecological significance and medicinal promise positions *P. oceanica* at the forefront of marine phytotherapy research, illustrating the intrinsic link between preserving marine biodiversity and discovering novel natural therapeutics (Figure 2).

This review aims to summarize the current understanding of *P. oceanica* health potential, detailing its molecular bioactive properties and potential therapeutic applications using the latest in vitro and in vivo studies. By bridging traditional uses and modern scientific insights, we seek to underscore the value of *P. oceanica* not only as a cornerstone of Mediterranean marine ecosystems but also as a valuable resource for future phytotherapeutic innovations. In doing so, we emphasize the imperative for integrated conservation and sustainable management strategies to ensure the long-term preservation and responsible utilization of this marine resource for future generations.

To achieve this, we conducted a systematic review of the scientific literature to evaluate the biomedical potential of *Posidonia oceanica* (L.) Delile, focusing on its phytochemical profile, bioactive properties, and possible therapeutic applications. Searches were performed in major scientific databases, including PubMed, Web of Science, Scopus, and Google Scholar, covering publications up to December 2025. Keywords used included combinations such as “*Posidonia oceanica*”, “bioactive compounds”, “marine phytotherapy”, “marine natural products”, “antioxidant”, “anti-inflammatory”, “anticancer”, “nanoparticle delivery”, “metabolic disorders”, and “antimicrobial activity”. Inclusion criteria encompassed peer-reviewed original research articles, reviews, and preclinical studies (both in vitro and in vivo) evaluating the biological activities or therapeutic potential of *P. oceanica*.

Schematic illustrations included in this review were created using the BioRender platform (www.biorender.com, University of Florence-BioMedical Science’s Plan) to visually represent the mechanisms of action and bioactive properties of *P. oceanica*, thereby enhancing the clarity and comprehension of the concepts discussed.

## 2. *P. oceanica*: Cytotoxic and Antimigratory Properties in the Fight Against Cancer

Cancer remains one of the most pressing global health challenges, with an estimated 20 million new cases and approximately 9.7 million deaths annually, highlighting the urgent need for effective strategies to combat this disease [6]. Currently, two primary approaches are employed to address cancer: prevention measures aimed at reducing risk factors and early detection, as well as conventional anticancer therapies such as chemotherapy, radiotherapy, and targeted treatments. Despite these efforts, resistance to antitumor drugs has emerged as a significant obstacle, often leading to therapeutic failures and poor prognosis for patients with malignant tumors [7,8]. Overcoming drug resistance remains a critical focus for ongoing research to improve treatment efficacy and patient outcomes worldwide.

In this context, natural plant-based products are emerging as promising adjuvant candidates for conventional therapies, owing to their wide-ranging actions that can boost treatment effectiveness or mitigate adverse side effects [9,10,11,12].

In the last ten years, the marine plant *P. oceanica* has garnered increasing interest in the field of pharmacological research, proving to be a promising resource with therapeutic potential [13].

In cancer research, the study of cell migration is of particular interest because the spread and dissemination of cancer in the body are the main causes of death in cancer patients [14]. In this context, the pioneering study by Barletta et al. (2015) [15] investigated, for the first time, the role of *P. oceanica* in relation to cancer cell migration and invasiveness. In particular, the study focused on a hydroalcoholic extract obtained from *P. oceanica* leaves (POE), which is mainly characterized by phenolic compounds such as catechins, epicatechins, gallic acid, chlorogenic acid, and ferulic acid. The authors demonstrated that POE is capable of inhibiting the migration and invasiveness of human fibrosarcoma (HT1080) cells by suppressing the expression and activity of matrix metalloproteinases (MMP-2 and MMP-9), which are crucial enzymes in tumor invasive processes. MMP-2 and MMP-9, also known as gelatinases, are involved in almost all forms of cancer, and their activity is associated with metastatic and invasive phenomena in various tumor types. For this reason, gelatinases represent a very promising therapeutic target against cancer [16]. In this context, the present findings are particularly relevant, as inhibition of gelatinases could impair cell migration and invasion, suggesting a potential migrastatic role for *P. oceanica*.

Further research conducted by Leri et al. (2018) [17] on the molecular mechanisms through which POE exerts anti-migratory properties has shown that POE acts by activating autophagy processes in HT1080 cells, with peak activation reached after approximately 7 h of cellular treatment. Scientific literature indicates that autophagy can indeed counteract the onset of cancer in its early stages of formation [18]. The ability of POE to activate early autophagic processes suggests a protective pathway against cancer by modulating vital cellular pathways (such as MAPKs and PI3K-AKT pathways), thus contributing to prevention and limitation of tumor progression.

The efficacy of POE in counteracting tumor cell migration was further observed in human neuroblastoma (SH-SY5Y) cells, confirming its migrastatic potential [19]. This study also demonstrated for the first time that POE promotes a long-term (5 days of treatment) neurite formation and the expression of mature neuronal markers in SH-SY5Y cells. It is well known that promoting differentiation in tumor cells can help to limit their invasive behavior [20]. Therefore, POE-induced morphological changes toward cell differentiation may, over time, enhance the anti-migratory effects of the phytocomplex in the tumor microenvironment.

Aqueous extracts of green and brown leaves and rhizomes of *P. oceanica* were studied by Abruscato et al. (2023) [21] to evaluate their potential cytotoxic effects on hepatocellular carcinoma (HepG2) cells. The differences in the chemical composition of the extracts influenced the observed effects: the rhizome extract (RE) contained higher levels of polyphenols, including delphinidin-3-glucoside and quercetin 3-O-galactoside, while it had lower concentrations of vanillic acid and proanthocyanidins B2 and B3. In the extracts from green (GLE) and brown (BLE) leaves, the most abundant polyphenols were, respectively, the methyl ester of caffeic acid and p-hydroxybenzoic acid. The results showed that 24-h exposure of the cells to the GLE and RE dose-dependently reduced the number of tumor cells, as well as inhibiting cell motility and long-term replication capacity, with a more pronounced effect observed for the RE. In contrast, BLE failed to show any biological effects at all tested doses. The identified mechanisms of action of GLE and RE included negative regulation of autophagy, induction of apoptosis, reduction in reactive oxygen species (ROS), and mitochondrial dysfunction in HepG2 cells. These findings suggest the therapeutic potential of different *P. oceanica* extracts, with their variable chemical characteristics influencing biological activities [21].

A study conducted by Punginelli et al. (2023) [22] investigated the cytotoxic role of fractions enriched with peptides extracted from the rhizomes and green leaves of *P. oceanica*. In particular, the natural peptide GEFALCSAKT (#7) and its derivative (#7d), obtained from the rhizome extract, as well as the derivative peptide NVVEL-NVAPGDK (#3d), obtained from the green leaf extract, showed a dose-dependent decrease in cell viability after 24 h of exposure and the ability to induce apoptosis in HepG2 cells. Therefore, the results obtained open new and diverse potential scenarios for future biomedical applications of the peptides identified from *P. oceanica*. These new peptides could constitute a promising chemical platform for the development of innovative therapies in the oncology field.

Falemban et al. (2025) [23] investigated the bioactive potential of *P. oceanica* leaf extracts, focusing on their anticancer properties. The authors identified several polyphenolic compounds via GC-MS analysis, including phenols and prostaglandins, such as 2,2′-methylenebis [6-(1,1-dimethylethyl)-4-methyl], prostaglandin A1-biotin, tris(2,4-di-tert-butylphenyl) phosphate, and desmetilverapamil. Results showed that *P. oceanica* extract exhibited potent cytotoxic effects against breast cancer (MCF-7) and hepatocellular carcinoma (HepG2) cells, surpassing the efficacy of conventional drugs like sorafenib and erlotinib. Molecular docking studies confirmed strong binding affinities of the phenolic compounds to key cancer-related kinases, such as EGFR T790M and VEGFR-2, which are enzymes involved in cancer progression. These findings support their potential as multi-target therapeutic agents.

Overall, *P. oceanica* demonstrates a multifaceted potential as a natural source of bioactive agents, particularly through its ability to inhibit tumor cell migration, induce apoptosis, promote differentiation, and modulate key molecular pathways involved in cancer progression (Table 1).

**Figure 3:**
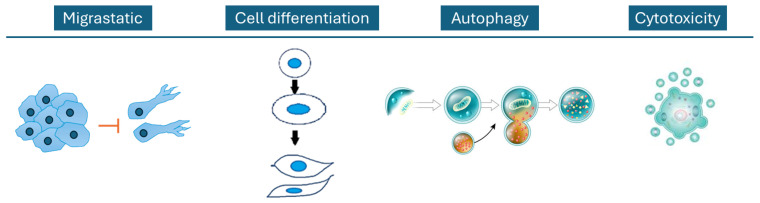
Schematic representation of the main mechanism of action of *P. oceanica* described in the fight against cancer.

The various phytocomplexes of *P. oceanica* containing diverse bioactive compounds, including peptides and polyphenols, exhibit significant anticancer and antimigratory effects across various cancer models (Figure 3). These promising findings highlight the importance of further research to fully elucidate the mechanisms involved and to develop *P. oceanica*-derived compounds into effective adjuvant or standalone treatments in oncology, offering hope for more targeted, less resistant, and potentially less toxic cancer therapies in the future.

## 3. Exploring the Antioxidant and Anti-Inflammatory Benefits of *P. oceanica*

Historical records and folk remedies have documented the *P. oceanica* use in treating a variety of ailments, including inflammatory conditions, skin disorders, and respiratory issues. Contemporary scientific research has increasingly focused on identifying the bioactive compounds responsible for these traditional effects, particularly its antioxidant and anti-inflammatory properties. These properties make *P. oceanica* a promising natural resource for the development of innovative solutions for health, skin care and inflammation management. This section delves into the scientific evidence supporting the antioxidant and anti-inflammatory benefits of *P. oceanica*, highlighting recent advances that underscore its potential in promoting human health and combating oxidative stress and inflammation-related diseases.

### 3.1. Antioxidant Effects of P. oceanica Extracts in Skin Health and Anti-Aging Applications

The increasing emphasis on antioxidant compounds in skincare highlights their vital role in combating oxidative stress, a key contributor to skin aging, photo-damage, and pigmentary disorders [24,25,26]. Oxidative stress arises from an imbalance between ROS production and endogenous antioxidant defenses, leading to collagen degradation, cellular damage, and dysregulation of melanogenesis. In this context, extracts from *P. oceanica* have garnered growing scientific interest over the past decade due to their rich polyphenolic composition, and associated antioxidant properties. *P. oceanica* extracts are particularly abundant in phenolic acids and flavonoids, which are widely recognized for their free radical scavenging activity and their ability to modulate redox-sensitive cellular pathways. These antioxidant-related properties contribute to the protection of skin cells from oxidative damage and to the maintenance of skin homeostasis. 

For instance, Cornara et al. (2018) [27] demonstrated that a hydroalcoholic (60% ethanol *v*/*v*) *P. oceanica* extract (PEE) exhibited marked antioxidant capacity, as quantified by the DPPH radical scavenging assay (IC_50_ = 32 ± 2 μg/mL), together with a high total polyphenol content. HPLC-MS analysis identified chicoric acid as the major compound, along with several flavonoids, supporting the role of this phytocomplex in counteracting oxidative stress. In the same study, PEE was shown to enhance the growth of human dermal (46BR.1N) fibroblasts and stimulate collagen synthesis, effects that are consistent with the ability of antioxidants to preserve fibroblast functionality and extracellular matrix homeostasis under oxidative conditions [27]. Additionally, PEE also exhibited dose-dependent inhibition of mushroom tyrosinase activity and a significant reduction in melanin production in melanoma (MeWo) cells. The relationship between these depigmenting effects and the antioxidant properties of PEE can be explained by the well-established role of ROS in melanogenesis. Oxidative stress is known to upregulate tyrosinase activity and melanogenic signaling pathways; therefore, the strong radical scavenging activity of PEE may contribute to limiting melanogenesis by reducing ROS levels and modulating redox-sensitive pathways involved in melanin synthesis. This suggests that the anti-tyrosinase and anti-melanogenic effects observed for PEE may be, at least in part, related to its antioxidant-rich polyphenolic composition, including chicoric acid. PEE also showed lipolytic effects on subcutaneous human primary preadipocytes, implying benefits for reducing cellulite and fat accumulation, likely due to its high chicoric acid content, known for antioxidant and anti-inflammatory properties [27]. 

Further evidence supporting the antioxidant potential of *P. oceanica* extracts was provided by Messina et al. (2021) [28], who showed that the phenolic profile of the leaves varies with their physiological stage. Green, photosynthetically active leaves displayed higher polyphenol content and stronger antioxidant potential compared to brown leaves. Optimized extraction methods, such as drying green leaves at 60 °C, yielded extracts (Gd-E) with high polyphenol content capable of improving cell viability post-UV exposure in human skin (HS-68) fibroblasts, underscoring their potential in skin protection and anti-aging formulations. 

Overall, these findings suggest that *P. oceanica* extracts exert well-documented antioxidant effects, which support skin health by neutralizing free radicals, protecting dermal cells from oxidative and UV-induced damage, promoting collagen synthesis, and indirectly modulating melanogenesis through redox regulation (Table 2). Overall, *P. oceanica* emerges as a promising natural marine phytocomplex for innovative skincare solutions aimed at preventing skin aging, reducing hyperpigmentation, and improving skin vitality.

### 3.2. Anti-Inflammatory Potential of P. oceanica: Experimental Evidence Across Inflammatory Models

The anti-inflammatory potential of *P. oceanica* has garnered increasing scientific interest, particularly as a promising alternative natural compound to conventional pharmacological treatments that often entail undesirable side effects. Inflammation, although a critical component of the immune defense, can contribute to the onset and progression of various chronic diseases when dysregulated. In this context, bioactive compounds derived from natural sources offer a compelling therapeutic avenue [29,30,31].

Building on this premise, preclinical studies have investigated the cellular and molecular mechanisms underlying the anti-inflammatory activity of *P. oceanica*. Vasarri et al. (2020) [32] showed that the hydroalcoholic extract of *P. oceanica* leaves (POE), known for its high polyphenol content, provides a cytoprotective effect in murine RAW264.7 macrophages stimulated with lipopolysaccharide (LPS). POE effectively reduced cytotoxicity and modulated key inflammatory mediators, including nitric oxide (NO) and cyclooxygenase-2 (COX-2). At the molecular level, POE significantly influenced the NF-κB signaling pathway, a pivotal axis in the regulation of inflammation, by inhibiting the phosphorylation and activation of NF-κB as well as its upstream regulators, ERK1/2 and Akt. This resulted in the downregulation of pro-inflammatory gene expression, highlighting the POE potential as an in vitro modulator of inflammatory responses [32].

Similarly, to previous studies using LPS-stimulated RAW264.7 macrophages [32], Abruscato et al. (2025) [33] evaluated the anti-inflammatory and immunomodulatory potential of aqueous extracts from *P. oceanica* green leaves (GLE) and rhizomes (RE). Both extracts reduced NO production and downregulated iNOS, COX-2, and TNF-α expression, while selectively modulating NF-κB activation. RE, in particular increased IL-10 production and enhanced endocytic activity. This effect may be linked to the presence of vanillic acid, which can activate signaling pathway such as the host stimulator of interferon genes (STING), promoting both anti-inflammatory responses and phagocytic function. Moreover, RE triggered phosphorylation of p38, MAPK, AKT, and JNK, pathways known to support macrophage endocytosis and immunomodulation. In contrast, GLE induced IL-1β upregulation and reduced endocytic activity, potentially due to compounds like p-coumaric acid that stabilize the cell membrane and limit phagocytosis. 

Molecular analyses revealed differential modulation of MAPKs and AKT; in particular, both extracts increased pJNK and pAKT levels, GLE prominently reduced pERK and RE strongly activated p38 MAPK, consistent with augmented macrophage functional responses. These effects are likely associated with the polyphenolic profiles of the extracts, including caffeic acid, caffeic acid methyl ester, catechin, p-coumaric acid, vanillic acid, and ellagic acid, as well as specific water-soluble proteins identified in proteomic analyses. Overall, these results highlight the capacity of *P. oceanica* extracts to finely regulate inflammatory pathway and macrophage activity, reinforcing their potential as natural modulators of immune responses [33].

Extending the investigation beyond immune cells, Abruscato et al. (2025) [34] have also explored the protective effects of *P. oceanica* acqueous extracts on endothelial cells, particularly at the blood–brain barrier (BBB), where neuroinflammation plays a key role in barrier dysfunction. *P. oceanica* extracts from green leaves (GLE) and rhizomes (RE) reduce TNF-α-induced NO production in brain-like endothelial cells (BLECs) co-cultured with human brain pericytes (hBPs), inhibit NLRP3 inflammasome activation, downregulate ICAM-1/VCAM-1, and preserve CLAUDIN-5 and VE-CADHERIN localization supporting BBB integrity. These protective effects are likely mediated by a synergistic combination of polyphenolic and antioxidant compounds. GLE, enriched in caffeic acid methyl ester, activates the HO-1/Nrf2 pathway to enhance endothelial resilience. RE, which contains delphinidin-3-glucoside, quercetin-3-O-galactoside, procyanidin dimers, vanillic acid, epicatechin, myricetin, ellagic acid, and trace resveratrol, contribute to anti-inflammatory and antioxidant activity. Mechanistically, these compounds reduce ROS generation, inhibit NF-κB and TLR4 signaling, upregulate junctional proteins, and support endothelial viability and barrier function [34].

Building on previous evidence of *P. oceanica* extracts modulating inflammatory responses in macrophages and brain-like endothelial cells, a recent study by Margheri et al. (2025) [35] highlighted the anti-inflammatory and antioxidant potential of hydroalcholic extract from *P. oceanica* leaves (POE) in human endothelial colony-forming cells (ECFCs). POE was shown to significantly reduce VEGF- and TNF-α-induced pro-inflammatory signaling, including the upregulation of adhesion molecules VCAM-1 and ICAM-1, without affecting cell viability. Mechanistically, the extract inhibited key pro-angiogenic pathways by stabilizing VEGFR2/KDR while decreasing its phosphorylation, and selectively attenuating downstream ERK and mTOR activation, thus impairing endothelial migration, invasion, and tube formation. Concurrently, POE reduced intracellular ROS levels and downregulated redox-sensitive genes (hTRX1, hTRX2, PRDX2, AKR1C1, AKR1B10), indicating a potent antioxidant action that contributes to modulation of inflammatory signaling. These effects are likely attributable to polyphenolic compounds such as chicoric and chlorogenic acids, consistent with earlier observations in RAW264.7 macrophages and BLEC-based BBB models. Collectively, these findings position *P. oceanica* as a multitarget natural modulator capable of limiting endothelial activation, oxidative stress, and inflammation, supporting its therapeutic potential in pathological conditions characterized by aberrant angiogenesis and vascular inflammation.

Consistent with these cellular-based experimental findings, the in vivo study conducted by Micheli et al. (2021) [36] on a CD-1 murine model of acute inflammatory pain confirms the potential of *P. oceanica* leaves extract (POE) as systemic anti-inflammatory agent. In this model, oral administration of POE (10–100 mg kg^−1^) exhibited dose-dependent effectiveness in reducing both inflammatory and oxidative markers, diminishing edema, and raising the pain threshold. Furthermore, POE markedly decreased myeloperoxidase (MPO) activity and tissue concentrations of inflammatory cytokines, including IL-1β and TNF-α, offering compelling evidence of its anti-inflammatory effects. Significantly, this study represents the first pharmacological evidence that orally administered *P. oceanica* can relieve inflammatory pain in an in vivo animal model [36].

Psoriasis is increasingly recognized as a chronic, immune-mediated systemic disease marked by widespread inflammation and characteristic skin lesions. Its pathogenesis centers on the dysregulation of immune pathways involving pro-inflammatory cytokines. Given its systemic nature, there is a pressing need for therapeutic approaches that can safely target these inflammatory mechanisms beyond the skin lesions. In this regard, a recent study by Micheli et al. (2024) [37] examined the effects of *P. oceanica* leaf hydroalcoholic extract (POE) in an in vivo C57BL/6 murine model presenting psoriasis-like skin lesions induced by Imiquimod (IMQ) for 5 days. Oral administration of POE (100 mg kg^−1^ for 5 days) significantly reduced the psoriasis area and severity index score and improved hallmark histological features, including hyperkeratosis. POE also markedly suppressed the tissue concentration of pro-inflammatory cytokines TNF-α, IL-17A, and IL-23, indicating its potential to modulate critical signaling pathways implicated in psoriasis, particularly the NF-κB pathway. Additionally, orally administrated POE lowered plasma levels of lipocalin-2, a promising biomarker and therapeutic target in psoriasis, further underscoring its systemic anti-inflammatory efficacy in the absence of toxicity in an in vivo animal model.

These findings, resumed in Table 3, underscore the significant therapeutic potential of *P. oceanica* as a safe and effective natural agent for managing chronic inflammatory conditions, paving the way for future clinical studies to fully explore its benefits in human inflammatory diseases.

**Figure 4:**
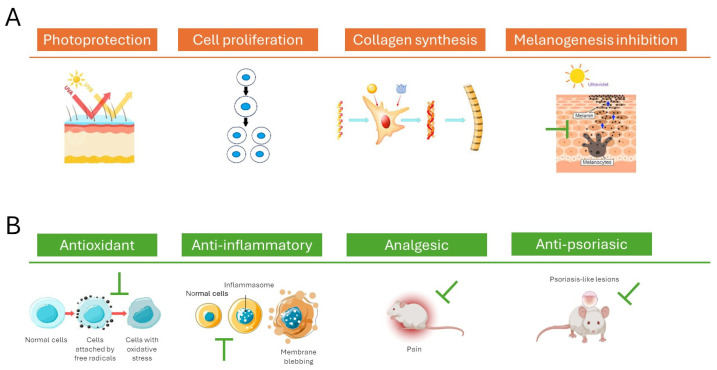
Schematic representation of the main mechanisms of action of *P. oceanica*, highlighting its (**A**) antioxidant activity for skin health and (**B**) anti-inflammatory benefits.

Overall, the findings from the literature on the multitarget biochemical mechanisms of action of *P. oceanica* (Figure 4) support the application potential of this marine plant as an innovative solution for skincare and anti-inflammatory benefits.

## 4. Glucose-Regulatory and Metabolic Effects of *P. oceanica* Extracts: Experimental Findings and Mechanistic Insights

*P. oceanica* has recently attracted scientific interest due to its potential therapeutic properties, particularly in the context of diabetes and its related complications. Traditional uses of this marine plant for managing metabolic disorders have prompted rigorous investigations into its biochemical effects. Several studies have explored the multifaceted actions of *P. oceanica* leaf extracts on glucose regulation, oxidative stress, vascular function, and lipid metabolism, highlighting its promise as a natural agent in diabetes management and associated comorbidities.

The pioneering study by Gokce et al. (2008) [38] demonstrated that oral administration of a hydroalcoholic extract (50% ethanol, *v*/*v*) from *P. oceanica* leaves in alloxan-induced diabetic rats lowers blood glucose levels in a dose-dependent manner. At higher doses (150 and 250 mg kg^−1^), the extract restores hepatic antioxidant enzyme activity, reduces lipid peroxidation and NO production in the liver, thereby supporting the protection of pancreatic β-cells. Notably, the lowest dose (50 mg kg^−1^) also reduced blood glucose but did not significantly influence oxidative stress markers, suggesting additional antidiabetic mechanisms beyond antioxidant effects. Diabetes-associated vascular dysfunction, characterized by impaired endothelial relaxation and increased vasoconstriction, is ameliorated by *P. oceanica* extract through improved endothelium-dependent vasorelaxation, likely mediated by activation of endothelial nitric oxide synthase (eNOS) via the PI3K pathway rather than by simply increasing NO bioavailability. Furthermore, the extract attenuates abnormal vascular contractions, probably through free radical scavenging and reduction in vasoconstrictor prostanoid formation, thereby exhibiting promising vasoprotective effects alongside glucose regulation [38].

One of the main complications of high blood glucose levels is the formation of advanced glycation end products (AGEs). These harmful compounds form when excess glucose reacts non-enzymatically with proteins, lipids, and nucleic acids, causing tissue damage and inflammation. AGEs contribute significantly to the progression of diabetic complications, including vascular dysfunction, kidney damage, and neuropathy. Controlling AGE formation is therefore a crucial target in managing diabetes and preventing its long-term effects. In this context, Vasarri et al. (2020) [39] reported that the hydroalcoholic extract from *P. oceanica* leaves (POE) significantly inhibits AGE formation in vitro, reducing fluorescence and electrophoretic mobility associated with glycation of human serum albumin (HSA). This anti-glycation activity, important for preventing and/or mitigating diabetic complications, appears to be partially independent of antioxidant mechanisms, underscoring the need for further mechanistic studies.

Building on this previous research, Morresi et al. (2022) [40] investigated the effects of *P. oceanica* hydroalcoholic extract (POE) on glucose metabolism using an in vitro model of human intestinal epithelial (Caco-2) cells. Their study demonstrated that POE reduces glucose absorption by downregulating the GLUT2 transporter without affecting SGLT1. Additionally, POE enhances intestinal barrier integrity and protects cells from oxidative stress, as evidenced by increased transepithelial electrical resistance and elevated levels of Zonula occludens protein. These findings suggest a potential role for POE in preventing intestinal dysfunction and inflammation associated with metabolic diseases.

Extending these observations from the intestine to the liver, recent in vitro evidence by Abruscato et al. (2025) [41] demonstrates that the glucose-regulatory effects of *P. oceanica* extracts are strongly dependent on the anatomical source of the plant material. Aqueous extracts from green leaves (GLE), but not from rhizomes, significantly enhanced glucose uptake and consumption in HepG2 cells. These effects were associated with activation of the IRS-1/AKT/PKCζ signaling pathway, increased GLUT-4 expression and membrane translocation, and concomitant downregulation of GLUT-2, indicating improved hepatic glucose handling. Proteomic analyses excluded the presence of insulin-like proteins, supporting a phytochemical-driven mechanism mainly attributable to polyphenols such as caffeic acid methyl ester. By improving hepatic glucose utilization at molecular level, these effects may contribute to limiting metabolic stress in hepatocytes [41].

Consistent with these glucose-related effects, alterations in hepatic metabolic homeostasis are tightly associated with lipid accumulation and the onset of non-alcoholic fatty liver disease (NAFLD), a condition that underscores the central role of the liver in integrating glucose and lipid metabolism. In NAFLD, excessive hepatic fat accumulation impairs glucose processing, thereby exacerbating insulin resistance and impairing glycemic control. Beyond its effects on glucose metabolism, *P. oceanica* exhibits beneficial actions on lipid metabolism by activating autophagy, a cellular mechanism essential for lipid degradation and NAFLD prevention. Vasarri et al. (2021) [42] showed that POE stimulates autophagic flux in human hepatocellular carcinoma (HepG2) cells, reducing lipid accumulation and cell viability, thereby indicating its potential to improve liver health.

Moreover, metabolic disorders characterized by elevated blood glucose levels are recognized risk factors for cancer. Lipid dysregulation plays a critical role in various types of cancer, including hepatocellular carcinoma, by promoting progression through the modulation of energy storage, metabolism, and cell signaling pathways. A well-established link exists between hepatic de novo lipogenesis and activation of the NF-κB pathway, which contributes to cancer metastasis through regulation of matrix metalloproteinases MMP-2 and MMP-9. POE has been shown to reduce lipid accumulation and fatty acid synthase expression under high glucose conditions in hepatocellular carcinoma (HepG2) cells, while also inhibiting the MAPK/NF-κB pathway and MMP-2/9 activities, suggesting promising adjuvant therapeutic potential in hepatocarcinoma management [43].

Overall, emerging scientific evidence highlights *P. oceanica* as a promising natural source with multiple biochemical mechanisms of action and multifaceted therapeutic potential for diabetes and metabolic disorders (Table 4).

**Figure 5:**
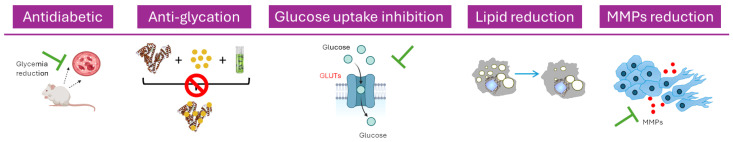
Schematic representation of the mechanisms of action of *P. oceanica* in glucose and lipid metabolism, based on in vitro and in vivo studies.

Bioactive compounds from *P. oceanica* exert a range of beneficial effects which contribute to improved glycemic control, lipid metabolism, and overall metabolic balance (Figure 5). These findings not only underscore the relevance of *P. oceanica* in the prevention and management of non-communicable diseases, but also support further investigation into its preclinical applications.

## 5. Innovative Delivery Systems for *P. oceanica* Extracts: Enhancing Bioavailability and Therapeutic Efficacy

The use of formulations to deliver plant extracts is a fundamental aspect in various sectors, including food, cosmetics, and pharmaceuticals [44]. Formulations play a crucial role in optimizing the absorption and bioavailability of the active ingredients contained in the extracts, while also ensuring stability and safety [45,46]. In the food sector, formulations may include advanced technologies such as nanoparticles or liposomes to deliver extracts of plants, vitamins, or minerals [47,48,49]. These innovations not only improve the solubility and absorption of nutrients but also prolong their shelf life while maintaining their organoleptic properties. In the pharmaceutical field, extract formulations are essential for the development of drugs with controlled and targeted release. Techniques such as microencapsulation protect the extracts from degradation, allowing for a gradual release in the body and enhancing therapeutic efficacy [50,51,52]. This innovative approach maximizes the potential of natural compounds, addressing the growing demands of consumers.

A significant example of these applications is provided by the study conducted by Piazzini et al. (2019) [53], which developed two distinct nanoformulations of the hydroalcoholic extract from the leaves of *P. oceanica* (POE), rich in polyphenols, using chitosan nanoparticles and Soluplus^®^ polymeric micelles. These formulations demonstrated promising potential in improving the solubility of POE, exhibiting favorable physical and chemical characteristics for parenteral administration, and maintaining excellent physical and chemical stability at 4 °C for three months. Notably, only the polymeric micelles showed a significant enhancement in the inhibitory activity of POE against the migration of human neuroblastoma (SH-SY5Y) cells. This marks an important step in enhancing a marine-origin phytocomplex, highlighting the significance of encapsulation within nanomicelles to improve solubility and bioactivity.

Additionally, recent research has analyzed the secondary metabolites of the rhizome of *P. oceanica* using UPLC-HRESI-MS/MS techniques, identifying as many as 86 compounds, including phenolic acids, flavonoids, and their sulfate conjugates [54]. The butanolic extract of *P. oceanica* demonstrated significant antioxidant and antidiabetic properties in vitro. To maximize therapeutic efficacy, a robust delivery system was developed by encapsulating the extract in gelatin nanoparticles, protecting the active ingredients and controlling their release. Results from an untargeted metabolomic analysis using GC-MS showed a significant reduction in fasting blood glucose levels and an improvement in insulin levels in diabetic Wistar albino rats, suggesting that this de-livery method is more effective than the simple extract in regulating altered metabolic processes [54]. This study offers new perspectives on the potential of the butanolic extract of *P. oceanica* encapsulated in gelatin nanoparticles as a promising and effective antidiabetic therapy. These findings are summarized in Table 5.

**Figure 6:**
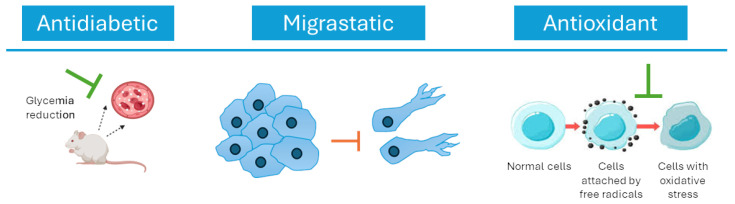
Schematic representation of the mechanisms of action of *P. oceanica* delivered through innovative formulations.

In summary, the application of nanocarrier technology to marine-origin phytocomplexes such as *P. oceanica* represents a novel strategy to maximize therapeutic efficacy while preserving extract integrity. These innovative delivery systems play a crucial role in maximizing the therapeutic potential of *P. oceanica* extracts by improving their bioavailability, stability, and targeted release (Figure 6). This advancement not only enhances the efficacy of natural compounds but also addresses key challenges in formulation science, thereby facilitating the development of more effective and reliable herbal-based therapies. Consequently, such progress supports the broader integration of herbal medicine into modern health and wellness practices, promoting its recognition as a valuable and scientifically grounded resource for preventive and therapeutic applications [55,56,57].

## 6. Antimicrobial and Antibiofilm Activities of *P. oceanica*: From Pathogen Control to Food Preservation

*P. oceanica*, a foundational seagrass of the Mediterranean Sea renowned for stabilizing seabeds and supporting diverse marine ecosystems, has emerged as a promising natural reservoir of bioactive compounds with potent antimicrobial properties. Despite hosting complex microbial communities on its leaves and rhizomes [58], *P. oceanica* demonstrates intrinsic antibacterial and antibiofilm activity of secondary metabolites, which is particularly significant in the context of escalating global antibiotic resistance.

Recent studies have shown that ethanolic and methanolic extracts from rhizomes of *P. oceanica* exhibit substantial antibacterial effects against reference strains and clinical isolates of *Enterococcus faecalis*, *Staphylococcus aureus*, *Escherichia coli*, and *Klebsiella pneumoniae*, including drug-resistant *S. aureus* strains from medical device infections. The rhizome extracts display minimum inhibitory concentrations (MICs) in the range of 0.2–0.5 mg/mL against Gram-positive strains, indicating strong potency, and show synergistic or additive effects when combined with ciprofloxacin, allowing lower effective doses against resistant strains. Notably, these extracts also disrupt biofilm formation by *S. aureus* and *E. faecalis* at concentrations similar to their MICs, with the ethanolic rhizome extract (ER) being the most effective [59].At the molecular level, advanced profiling techniques such as nanoRP-UHPLC and HRMS have led to the identification of nine peptides within *P. oceanica* acid acetic extracts from green leaves and rhizomes [22]. Among these, natural peptide #3 (NVVELNVAPGDK) exhibited strong biofilm inhibition against *E. coli* and *S. aureus*. Synthetic derivative peptides were designed using bioinformatic optimization to improve stability, selectivity, and antimicrobial properties; however, most derivatives displayed limited activity in vitro. Notably, peptide #5d (IVASVGSA) showed notable antibiofilm activity against *Pseudomonas aeruginosa*, likely due to enhanced physicochemical properties [22]. These findings highlight that natural and rationally designed peptides from *P. oceanica* may serve as promising antimicrobial agents, although their efficacy is strongly peptide- and target-dependent. 

Beyond potential clinical applications, *P. oceanica* extracts have demonstrated utility in food preservation [60]. When applied to fresh-cut fruits like peaches, they reduce microbial spoilage and extend shelf life by inhibiting fungal pathogens such as *Aspergillus niger* and *Penicillium chrysogenum*. This effect is attributed to the high phenolic content and antioxidant activity of the extracts, which delay oxidative degradation. Minimum inhibitory concentrations (MICs) for the tested *P. oceanica* extracts were reported at 2 g/L against Gram-positive bacteria, consistent with their observed antifungal activity [60]. These results are summarized in Table 6.

**Figure 7:**
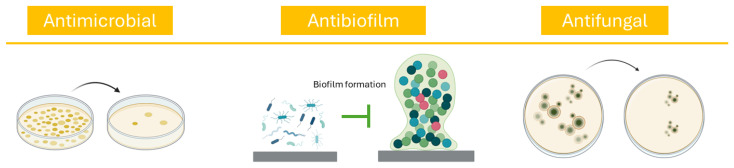
Schematic representation of the antimicrobial, antibiofilm, and antifungal properties of *P. oceanica*.

Altogether, the accumulating evidence underscores *P. oceanica* as a potent source of multifunctional antimicrobial agents, capable of addressing drug-resistant infections and enhancing food safety (Figure 7). Continued investigation into its chemical composition and mechanisms of action will be critical for unlocking its full adjuvant potential in therapeutic and industrial applications.

## 7. Enzymatic Inhibitory and Neuroprotective Activities of *P. oceanica*

Marine plants are increasingly recognized as sustainable and versatile sources of bioactive compounds with potential applications in human health. Among them, *P. oceanica* has attracted attention not only for the previous described bioactivities, but also for its potential as a natural multitarget inhibitor of key enzymes implicated in neurodegeneration, metabolic regulation, and microbial pathogenicity. Enzymatic modulation represents a strategic approach for the prevention and management of disorders such as Alzheimer’s disease, urease-related infections, and other enzyme-driven pathologies. In this context, recent study has explored the capacity of *P. oceanica* extracts to inhibit enzymes such as acetylcholinesterase (AChE), and butyrylcholinesterase (BChE) supporting their potential neuroprotective and therapeutic relevance.

A recent study by Karima et al. (2025) [61] investigated the enzymatic inhibitory activity of methanolic extracts of *P. oceanica* leaves and rhizomes collected from the eastern Algerian coast. Using LC-MS/MS analysis, 23 and 22 secondary metabolites were identified in the leaf and rhizome extracts, respectively, including phenolic compounds such as polydatin, rutin, and vanillin. The extracts were evaluated for their inhibitory activity against AChE and BChE using standard colorimetric assays. The leaf extract showed IC_50_ values of 113.43 ± 0.77 µg/mL against AChE, and 30.34 ± 0.56 µg/mL against BChE. The rhizome extract exhibited IC_50_ values of 33.59 ± 0.73 µg/mL (AChE), and 11.82 ± 0.73 µg/mL (BChE). These results indicate comparable or even superior inhibitory activity relative to standard reference compounds such as galantamine (for cholinesterases).Molecular docking studies supported these findings, demonstrating strong binding interactions between specific phenolic compounds and the active sites of the target enzymes. Polydatin displayed high affinity for AChE (−7.765 kcal/mol), and rutin showed the strongest binding to BChE (−9.533 kcal/mol). Importantly, cytotoxicity assays on Vero cells (continuous cell line derived from the kidney of an African green monkey) revealed moderate toxicity only at high concentrations, confirming a favorable safety margin for effective enzymatic inhibition [61].

Beyond cholinesterase inhibition, *P. oceanica* phenolics may potentially contribute to neuroprotection through additional mechanisms, including antioxidant activity that reduces oxidative stress, and anti-inflammatory effects that may mitigate neuroinflammation. Some compounds may also modulate neuroprotective signaling pathways, indirectly supporting neuronal survival. Although direct pharmacokinetic data on *P. oceanica* extracts are limited, the physicochemical properties of certain phenolics, such as vanillin and polydatin, suggest a potential for adequate solubility and bioavailability to exert effects in vivo, warranting further investigation.

These findings highlight the potential of *P. oceanica* as a natural source of multitarget enzyme inhibitors with applications in neuroprotection (Table 7). The study also underscores the relevance of phenolic compounds as key contributors to the observed bioactivities, reinforcing the value of marine plants as sustainable therapeutic resources.

## 8. Conclusion and Future Directions

Over the past decade, *P. oceanica* has evolved from being recognized solely for its ecological importance in the Mediterranean basin to gaining scientific attention as a valuable reservoir of bioactive compounds with broad therapeutic potential. While historically utilized for its physical properties and minor traditional remedies, modern research has unveiled a diverse phytochemical profile in *P. oceanica*, notably polyphenols peptides, polysaccharides, and other secondary metabolites capable of exerting significant biomedical effects across various domains.

**Figure 8:**
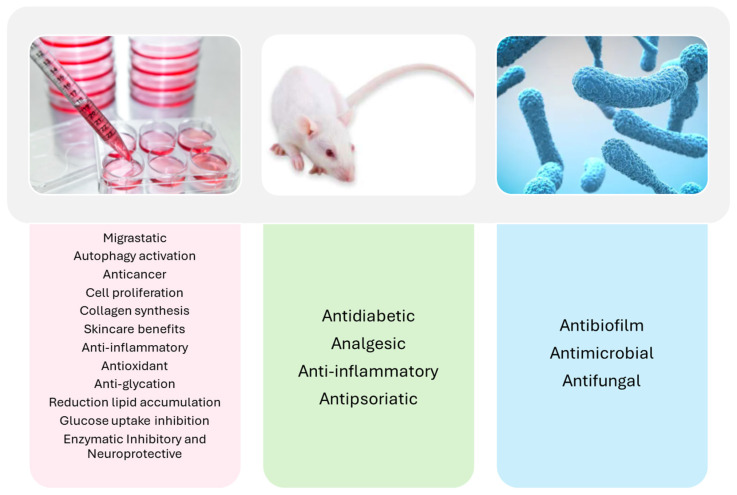
Schematic overview of the emerging bioactive properties of *P. oceanica* extracts demonstrated in different experimental models: in vitro cell-based assays (red box), in vivo animal models (green box), and in vitro bacterial and fungal assays (blue box).

Accumulating experimental data demonstrate that different *P. oceanica* extracts possess anticancer and anti-migratory properties, particularly relevant in the oncology field. These effects are mediated through molecular mechanisms such as apoptosis induction, autophagy regulation, and inhibition of key enzymes, such as MMPs. Simultaneously, *P. oceanica* antioxidant and anti-inflammatory activities support potential applications in dermatological conditions, including psoriasis, and inflammatory disorders more broadly. In metabolic health, evidence indicates that *P. oceanica* extracts contribute to glucose-lowering, anti-glycation, and lipid-modulating effects, making them promising candidates for managing type 2 diabetes and associated complications like non-alcoholic fatty liver disease. Importantly, recent advancements in nanocarrier technologies, including liposomes, nanoparticles, and micelles, have significantly improved the bioavailability, stability, and targeted delivery of *P. oceanica* crude extracts, facilitating their integration into pharmaceutical and cosmeceutical formulations. Additionally, its antimicrobial and antibiofilm activities present novel strategies for addressing antibiotic resistance and enhancing food preservation (Figure 8).

It is important to note that since 2008, the year the first scientific evidence supporting the traditional uses of *P. oceanica* was published, research interest in this marine plant has steadily grown over the past decade, as demonstrated by the scientific articles included in this review. Looking ahead, *P. oceanica* stands as a compelling example of the emerging potential of marine phytotherapy, a field that is increasingly aligned with the global shift toward sustainable, plant-based, and nature-derived health interventions. When sourced through responsible and environmentally conscious practices, marine-derived therapeutics uphold the principles of ecological stewardship. The development of several marine-derived compounds as approved drugs highlights the potential of the marine ecosystem as a rich source of new drugs [62,63].

Nevertheless, while current preclinical studies provide promising evidence supporting the biomedical potential of *P. oceanica*, these findings must be rigorously validated through detailed mechanistic studies and well-designed clinical trials to ensure reproducibility, safety, and therapeutic efficacy. Ultimately, *P. oceanica* exemplifies how marine biodiversity can be harnessed to develop sustainable, science-based health solutions. With ongoing interdisciplinary research and sustainable utilization strategies, this Mediterranean endemic seagrass holds the potential to become a pivotal contributor to the next generation of marine phytotherapeutic innovations in modern medicine promoting human health while ensuring the preservation of marine ecosystems.

### Safety Considerations

While numerous preclinical studies in vitro and in vivo (oral administration in mice) indicate that *P. oceanica* extracts exert beneficial biological effects without detectable toxicity, it is important to highlight that no clinical studies in humans have been reported to date. Additionally, the biological activity and safety profile of *P. oceanica* can be influenced by the heterogeneity of the extracts, including differences in plant parts, extraction methods, concentrations used, and molecular targets. Some studies have shown that aqueous extracts may exert cytotoxic effects on tumor cells; however, the current literature does not report compounds from *P. oceanica* as toxic to humans. These considerations emphasize the need for careful characterization of extracts and well-designed clinical trials before translation to human applications.

## Figures and Tables

**Table 1:** Bioactive Compounds from *P. oceanica* as Natural Anticancer Agents: From Cell Viability to Migration Inhibition.

Plant Material	Extraction Method	Compound Class	Experimental Model	Bioactivity	Reference
Green leaves	Hydroalcoholic	Polyphenols	Human Fibrosarcoma (HT1080) cells	Migrastatic	[15]
Green leaves	Hydroalcoholic	Polyphenols	Human Fibrosarcoma (HT1080) cells	Autophagy activation	[17]
Green leaves	Hydroalcoholic	Polyphenols	Human Neuroblastoma (SH-SY5Y) cells	Migrastatic; Cellular differentiation	[19]
Green and Brown leaves; Rhizome	Aqueous	Polyphenols	Hepatocellular carcinoma (HepG2) cells	Cytotoxicity	[21]
Green; Rhizome	Acetic acid	Peptides	Hepatocellular carcinoma (HepG2) cells	Cytotoxicity	[22]
Green leaves	Aqueous	Polyphenols	Breast cancer (MCF-7) and Hepatocellular carcinoma (HepG2) cells	Cytotoxicity	[23]

**Table 2:** Properties of *P. oceanica* in skin health.

Plant Material	Extraction Method	Compound Class	Experimental Model	Bioactivity	Reference
Green leaves	Hydroalcoholic	Polyphenols	Stabilized human dermal fibroblasts (46BR.1N); human melanoma (MeWo) cells; subcutaneous human primary preadipocytes	Cell proliferation; collagen synthesis; tyrosinase activity inhibition; reduction in melanin production; lipolytic effects	[27]
Green leaves	Hydroalcoholic	Polyphenols	Human skin fibroblast (HS-68)	Photoprotective role	[28]

**Table 3:** Anti-Inflammatory Properties of *P. oceanica* extracts.

Plant Material	Extraction Method	Compound Class	Experimental Model	Bioactivity	Reference
Green leaves	Hydroalcoholic	Polyphenols	murine macrophages (RAW264.7)	Cytoprotective activity; anti-inflammatory activity	[32]
Green leaves	Aqueous	Polyphenols; water soluble proteins	murine macrophages (RAW264.7)	Anti-inflammatory activity; immunomodulatory activity	[33]
Rhizomes	Aqueous	Polyphenols; water soluble proteins	murine macrophages (RAW264.7)	Anti-inflammatory activity; immunomodulatory activity; stimulation of macrophage phagocytosis	[33]
Green leaves, and rhizomes	Aqueous	Polyphenols	Human BLECs + hBPs (in vitro BBB model)	Anti-inflammatory activity; antioxidant activity; barrier-protective effect	[34]
Green leaves	Hydroalcoholic	Polyphenols	human endothelial colony-forming cells (ECFCs)	Anti-inflammatory activity; Antioxidant activity; Anti-angiogenic activity; Anti-migratory activity; Anti-invasive activity	[35]
Green leaves	Hydroalcoholic	Polyphenols	CD-1 mice	Anti-inflammatory activity; antioxidant activity; anti-edematous effect; analgesic activity	[36]
Green leaves	Hydroalcoholic	Polyphenols	C57BL/6 mice	Anti-inflammatory: Anti-psoriasic	[37]

**Table 4:** Bioactivities of *P. oceanica* in Glucose Regulation and Metabolic Disorders.

Plant Material	Extraction Method	Compound Class	Experimental Model	Bioactivity	Reference
Green leaves	Hydroalcoholic	Polyphenols	alloxan-induced Wistar albino diabetic rats	Antidiabetic	[35]
Green leaves	Hydroalcoholic	Polyphenols	in vitro HSA	anti-glycation	[36]
Green leaves	Hydroalcoholic	Polyphenols	human intestinal epithelial (Caco-2) cells	Antioxidant; Reduction in glucose uptake	[37]
Green leaves	Aqueous	Polyphenols	hepatocellular carcinoma (HepG2) cells	Anti-hyperglycemic activity; glucose uptake and consumption enhancement; GLUT-4 upregulation; GLUT-2 downregulation	[41]
Green leaves	Hydroalcoholic	Polyphenols	hepatocellular carcinoma (HepG2) cells	Reduction in lipid accumulation; autophagy activation	[42]
Green leaves	Hydroalcoholic	Polyphenols	hepatocellular carcinoma (HepG2) cells	Reduction in lipid accumulation; reduction in MMPs	[43]

**Table 5:** Bioactive Formulations of *P. oceanica* extracts.

Plant Material	Extraction Method	Compound Class	Experimental Model	Bioactivity	Reference
Green leaves	Hydroalcoholic	Polyphenols	Human neuroblastoma (SH-SY5Y) cells	Migrastatic	[53]
Rhizome	Methanolic	Polyphenols	diabetic Wistar albino rats	Antioxidant; antidiabetic	[54]

**Table 6:** Antimicrobial Potentials of *P. oceanica* Extracts.

Plant Material	Extraction Method	Compound Class	Strain	Bioactivity	Reference
Rhizome	hydroalcoholic and methanolic		*E. faecalis*; *S. aureus*; *E. coli*; *K. pneumoniae*	Anti-microbial; antibiofilm	[59]
Green leaves and rhizomes	Acid acetic	Peptides	*E. coli*, *S. aureus*, *P. aeruginosa*	antibiofilm	[22]
Green leaves	hydroalcoholic	Polyphenols	*A. niger*, *P. chrysogenum*	antifungal	[60]

**Table 7:** Enzymatic/neuroprotective bioactivity of *P. oceanica* extracts.

Plant Material	Extraction Method	Compound Class	Experimental Model	Bioactivity	Reference
Green leaves, and rhizomes	Methanolic	Polyphenols	In vitro enzymatic assays (AChE, BChE); Cytotoxicity on Vero cells	Anti-AChE, Anti-BChE Moderate cytotoxicity	[61]

## Data Availability

No new data were created or analyzed in this study. Data sharing is not applicable to this article.

## Abbreviations

The following abbreviations are used in this manuscript:

AChE	Acetylcholinesterase
AGEs	Advanced glycation end-products
AKR1B10	Aldo-Keto Reductase Family 1, Member B1
AKR1C1	Aldo-Keto Reductase Family 1, Member C1
AKT	Protein Kinase B (AKT)
BBB	Blood–Brain Barrier
BChE	Butyrylcholinesterase
COX-2	Cyclooxygenase-2
EGFR T790M	Epidermal Growth Factor Receptor with T790M mutation
eNOS	endothelial Nitric Oxide Synthase
ERK1/2	Extracellular signal-Regulated Kinases 1 and 2
GC-MS	Gas Chromatography–Mass Spectrometry
GLUT2	Glucose Transporter Type 2
GLUT4	Glucose Transporter Type 4
HO-1	Heme Oxygenase-1
HPLC-MS	High-Performance Liquid Chromatography–Mass Spectrometry
HRMS	High-Resolution Mass Spectrometry
HSA	Human Serum Albumin
hTRX1	Human thioredoxin 1
hTRX2	Human thioredoxin 1
ICAM-1	Intercellular Adhesion Molecule 1
IL-17A	Interleukin 17A
IL-1β	Interleukin 1 beta
IL-23	Interleukin 23
IMQ	Imiquimod
iNOS	inducible Nitric Oxide Synthase
IRS-1	Insulin Receptor Substrate 1
JNK	c-Jun N-terminal kinase
KDR	Kinase insert Domain Receptor
LPS	Lipopolysaccharide
MAPKs	Mitogen-Activated Protein Kinases
MMP-2	Matrix Metalloproteinase-2
MMP-9	Matrix Metalloproteinase-9
MPO	Myeloperoxidase
mTOR	mechanistic Target Of Rapamycin
NAFLD	Non-Alcoholic Fatty Liver Disease
nano-RP-UHPLC	nano-Reverse Phase Ultra High-Performance Liquid Chromatography
NF-κB	Nuclear Factor kappa-light-chain-enhancer of activated B cells
NLRP3	NOD-, LRR- and pyrin domain-containing protein 3
NO	Nitric Oxide
Nrf2	Nuclear factor (erythroid-derived 2)-like 2
p38 MAPK	p38 Mitogen-Activated Protein Kinase
p-AKT	phosphorylated AKT
PERK	Protein kinase RNA-like Endoplasmic Reticulum Kinase
PI3K	Phosphoinositide 3-kinases
p-JNK	phosphorylated c-Jun N-terminal kinase
PKCζ	Protein Kinase C zeta
PRDX2	Peroxiredoxin 2
ROS	Reactive Oxygen Species
SGLT1	Sodium-Glucose Transporter 1
STING	Stimulator of Interferon Genes
TLR4	Toll-Like Receptor 4
TNF-α	Tumor Necrosis Factor alpha
UPLC-HR-ESI-MS/MS	Ultra-Performance Liquid Chromatography-High-Resolution Electrospray Ionization Tandem Mass Spectrometry
VCAM	Vascular Cell Adhesion Molecule
VCAM-1	Vascular Cell Adhesion Molecule 1
VE-cadherin	Vascular Endothelial cadherin
VEGFR-2	Vascular Endothelial Growth Factor Receptor 2
